# Diel-scale temporal dynamics recorded for bacterial groups in Namib Desert soil

**DOI:** 10.1038/srep40189

**Published:** 2017-01-10

**Authors:** Eoin Gunnigle, Aline Frossard, Jean-Baptiste Ramond, Leandro Guerrero, Mary Seely, Don A. Cowan

**Affiliations:** 1 Centre for Microbial Ecology and Genomics, Genomic Research Institute, Department of Genetics, University of Pretoria, South Africa; 2 Gobabeb Research and Training Centre, Walvis Bay, Namibia; 3 School of Animal, Plant and Environmental Sciences University of the Witwatersrand, South Africa

## Abstract

Microbes in hot desert soil partake in core ecosystem processes e.g., biogeochemical cycling of carbon. Nevertheless, there is still a fundamental lack of insights regarding short-term (i.e., over a 24-hour [diel] cycle) microbial responses to highly fluctuating microenvironmental parameters like temperature and humidity. To address this, we employed T-RFLP fingerprinting and 454 pyrosequencing of 16S rRNA-derived cDNA to characterize potentially active bacteria in Namib Desert soil over multiple diel cycles. Strikingly, we found that significant shifts in active bacterial groups could occur over a single 24-hour period. For instance, members of the predominant Actinobacteria phyla exhibited a significant reduction in relative activity from morning to night, whereas many Proteobacterial groups displayed an opposite trend. Contrary to our leading hypothesis, environmental parameters could only account for 10.5% of the recorded total variation. Potential biotic associations shown through co-occurrence networks indicated that non-random inter- and intra-phyla associations were ‘time-of-day-dependent’ which may constitute a key feature of this system. Notably, many cyanobacterial groups were positioned outside and/or between highly interconnected bacterial associations (modules); possibly acting as inter-module ‘hubs’ orchestrating interactions between important functional consortia. Overall, these results provide empirical evidence that bacterial communities in hot desert soils exhibit complex and diel-dependent inter-community associations.

Macro- and micro-environmental parameters are critical in governing community assembly and function^1^,^2^. This is especially true in hot desert ecosystems, where extreme environmental conditions have led to discrete adaptations (i.e., collectively leading to niche specialization) for many plants and animal species^3^,^4^,^5^. Such extreme conditions are also considered to be key drivers of both the structure and function of edaphic microbial communities^6^,^7^,^8^. Given that climate change models predict major shifts in future temperature and precipitation regimes for dry-land regions (e.g., 10–30% reduced precipitation in subtropical arid zones by the end of the 21st century^9^), changes in complex multi-trophic responses over short and long time scales are likely to impact significantly on core ecosystem processes (e.g., carbon storage in soils^10^). In consequence, ecologists have become increasingly interested in further understanding the influence of temporality in shaping community structures and functions in hot desert soils^11^,^12^,^13^, which in turn can aid in predicting future ecological impacts.

To date, research on microbial temporal dynamics in hot deserts has been largely focused on diversity patterns over seasonal and yearly scales^14^,^15^,^16^,^17^ and/or after a wetting event^18^,^19^,^20^. Although key insights into the causative factors (deterministic vs. stochastic) driving microbial community structure have been provided across biogeographic space^21^,^22^, comparatively little is known about the fine-scale temporal changes in edaphic microbial community structure and function. Indeed, other than studies focused on Cyanobacteria^23^,^24^ and a temperature manipulation experiment^25^, short temporal community dynamics have been neglected. This is surprising given the fact that significant differences in specific desert soil processes have been recorded over the diel (24 hrs) cycle (e.g., CO_2_ flux refs 26, 27, 28). In addition, asymmetrical diel warming; i.e., the increase in daily minimum temperature (T_min_), is widely observed and set to continue in terrestrial ecosystems^29^,^30^ and is likely to impact negatively on microbial communities and their processes^31^.

Since Noy-Meir^32^ highlighted the influence of pulsed water events in arid systems over 40 years ago, it has been widely believed that hot desert edaphic microbial communities remain relatively static during extended periods of dryness. Stemming from this, the modern pulse-reserve ‘bioavailable water’ paradigm^33^ proposes a restriction of biological activity in arid soils to pre- and post episodic rainfall events, although this response can vary considerably depending on the duration and intensity of these events^34^,^35^,^36^. By definition however, rainfall events are extremely infrequent in hot desert regions. For instance, fewer than 10 rainfall events occur annually in the Namib Desert^37^ and parts of the Atacama Desert may receive a rainfall event only once per decade^38^. Recent evidence has demonstrated that diverse microbial populations can be found under ‘standard’ conditions in both cold desert^39^,^40^ and hot desert soil^38^,^41^,^42^. Although many of these cells are likely to have been dormant, many organisms can endure the effects of a water-limited environment by employing specific adaptation mechanisms that include (i) the accumulation of compatible solutes (e.g., trehalose, sucrose) and/or (ii) the production of exopolysaccharides. This has been demonstrated for key edaphic bacterial phyla, such as Proteobacteria^43^, Actinobacteria^44^ and Cyanobacteria^23^. In coastal deserts, such as the Namib and Atacama Deserts, moisture inputs such as fog and dew precipitation are fundamental in sustaining hot desert insect and plant populations^45^,^46^, as well as microbial communities^47^,^48^,^49^,^50^,^51^. Fog events can occur when the water vapor concentration in the atmosphere reaches saturation, i.e., 100% relative humidity (RH)^52^. As temperature and relative humidity have an interdependent relationship, inverse maximum values are an important feature of hot desert systems over the diel cycle (day [temperature maximum] vs. night [RH maximum) (e.g., Atacama Desert ref. 52). These insights provide much scope to the possibility that although microbial communities are highly reactive to water pulse events (i.e., rainfall), microbial activity within the soil matrix may not be entirely limited to these timelines; particularly in the fog-impacted Namib Desert^37^.

Given the reduced microbial species richness and diversity in desert soils when compared to mesic-type soils^41^, this environment presents a model system in which to examine the ecology of *in situ* edaphic microbial consortia. As well as this, the improved access to modern high throughput sequencing and bioinformatics tools have greatly enhanced our capacity to interrogate the complexity of microbial community dynamics in soil^33^,^41^,^51^,^53^. For instance, network analysis can be used to evaluate inter- and intra- taxa associations through statistically robust co-occurrence patterns and theorize on species interactions (e.g., competitive, cooperative or null)^33^,^51^,^53^. Therefore, there is much scope in utilizing this comparatively less complex soil environment to answer pertinent ecological questions relating to microbial function and community dynamics.

In our experimental approach, we investigated the diversity (community composition) of potentially active bacterial communities (total RNA-based) in Namib Desert soil over multiple diel cycles. More specifically, we aimed to test the hypothesis that day-night variations in microclimatic factors (i.e., temperature and humidity) would reflect changes in active hot desert soil bacterial communities. We also aimed to determine if the recorded environmental factors were solely responsible for any observed structural shifts or did other factors (e.g., biotic factors such as microbial interactions) also play a role. In order to accomplish this we employed molecular fingerprinting (T-RFLP) together with 454 pyrosequencing of 16S rRNA gene amplicons. Community data analyses incorporated an array of physicochemical parameters together with climatic measurements recorded throughout the 5-day sampling period, which comprised of three daily sampling time points (morning, midday and night).

## Results and Discussion

The light-dark (diel) cycle drives behavioral responses that are an essential component of life in hot desert systems. In higher organisms, well-characterized circadian rhythms facilitate adaptive strategies within these habitats that include the reproductive behaviour of desert locusts^54^, heterothermy in desert mammals^55^ and in desert plant physiology^56^. It has also been reported that the globally distributed circadian gene locus, *cpm*A, is highly conserved in key microbial phylotypes such as Cyanobacteria^57^, which suggests a key role in the maintenance of intracellular homeostasis and adaptation^58^. For mixed communities, light-dependent changes in microbial gene expression are also a feature in hot desert biological soil crusts^23^. By focusing on RNA isolated from bacterial communities *in situ*, we provide evidence that changes in active bacterial groups can occur over fine time scales and that both environmental selection and biotic associations are crucial factors within this system.

### Site characteristics

**Figure 1:**
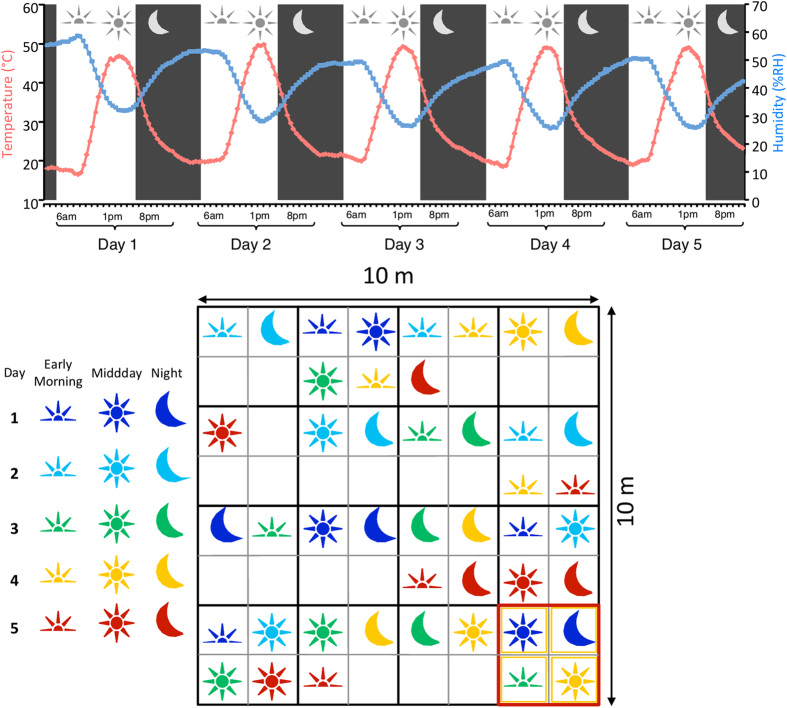
Experimental design and environmental parameters (temperature & humidity) measured over the 5-day study at a local site adjacent to the Gobabeb Training and Research Centre, Namib Desert.

No rainfall or fog events were recorded on site during the experimental period. Historically, fog events have provided a key source of bioavailable moisture in Gobabeb^37^. Fog events have an interdependent relationship with atmospheric humidity, which tends to reach maximum levels in the morning before dissipating quickly after sunrise^37^,^52^. During this study, *in situ* soil relative humidity (RH) levels were highly variable over each diel cycle (Fig. 1; time-points; P < 0.001). Soil RH values ranged from 25% (midday, day 4) to 59% (night, day 1). The diel RH profiles differed between days with the RH profile on day 1 was found to vary significantly from those of days 2, 3 and 4 (*post hoc* testing with Tukey-HSD comparisons; P < 0.001). An increase in peak soil temperature after day 1 (day 1, 47 °C, days 2 to 5, ≥50 °C) may have accounted for this shift in RH values. Physiocochemical parameters (pH, organic C, conductivity, mass concentrations of P, Ca, K, Mg, Na, S) were relatively homogenous over the entire sampling period (Table S1). Significant differences were found between days for nitrate (F_4.45_ = 3.54, P = 0.017) and magnesium (F_4.45_ = 3.01, P = 0.037) but this was likely to be a consequence of spatial heterogeneity, with a nutrient ‘hotspot’ recorded for a single biological replicate sampled on day 2 (Fig. S1).

### Changes in active bacterial communities revealed by T-RFLP analysis

**Figure 2:**
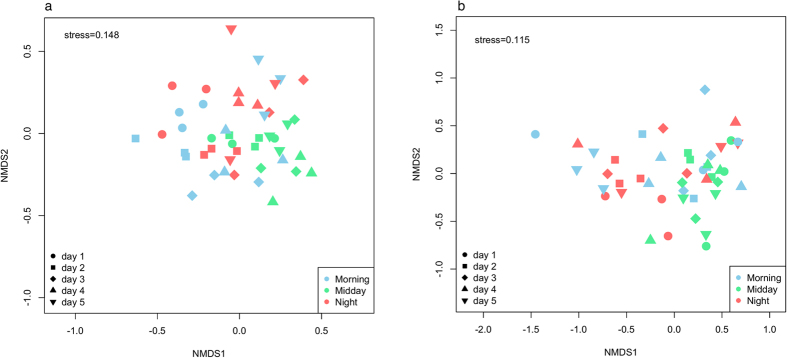
Non-metric multidimensional scaling (nMDS) ordination of Bray–Curtis distances between microbial communities (cDNA-based) inferred from (**a**) T-RFLP community fingerprinting and (**b**) 454-pyrosequencing. Each point represents a bacterial community at a specific time-point (n = 45).

Community fingerprinting by T-RFLP rRNA gene (cDNA) analysis recorded a total of 27 unique terminal restriction fragments (TRFs), likely representing the most active bacterial taxa present in this soil system^59^. The number of TRFs per sample (α-diversity) ranged from 12 to 23 with only 4 (15%) and 2 (7%) TRFs not detected for each day or time-point, respectively. Despite the absence of discernable clustering patterns for both Morning and Night bacterial communities through nMDS ordination, the Midday communities did cluster relative to the other time-points (Fig. 2a). Significant shifts in potentially active bacterial communities was recorded between time-points was confirmed by PERMANOVA (F_4,45_ = 5.51, P = 0.007). Interestingly, the T-RFLP community profiles were not found to be significantly different between days (F_4,45_ = 0.15, P = 0.961). This was also found in 454 pyrosequencing analysis using the same samples (Fig. 2b; time-points, F_4,44_ = 1.12, P = 0.04)

### Considerable diversity in hot desert soil bacterial communities

**Figure 3:**
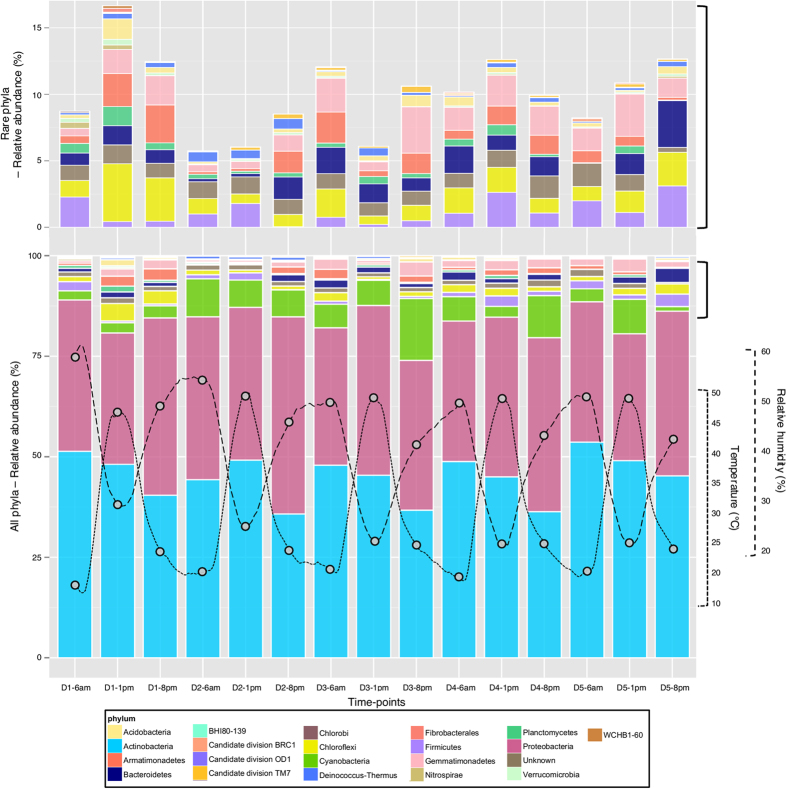
Color-coded bar plot showing the average bacterial phylum distribution in Namib Desert soil over five diel cycles. These patterns are overlayed with climatic data to give an adequate representation of temporal fluctuations within the bacterial groups present.

In order to provide further insight into these findings we performed deep-sequencing analysis. 454 pyrosequencing of 16S rRNA gene amplicons (generated from the same cDNA pool as for T-RFLP analyses) resulted in a total of 4348 OTUs (96 316 sequence reads, n = 45) at the 97% cutoff (cutoff level for all OTUs if not stated otherwise), with 1414 singletons (32%) and 606 doubletons (14%). After sub-sampling this dataset for sequence consistency (1702 OTUs > 15,000 reads), the number of singletons and doubletons increased to 47% and 15%, respectively. The large number of ‘rare’ OTUs identified here is not uncommon for hot desert soil communities^6^,^38^ and can represent novel diversities within the rare biosphere^60^. Alpha-diversity was relatively consistent between samples regardless of the diversity metric used; i.e., with no significant differences between days or time-points (Table 1). For many of the communities sampled, rarefaction analysis exhibited approximate plateau plot behavior (Figure S2), suggesting that sampling depth was sufficient to properly assess the bacterial diversity within this system. A total of 20 bacterial phyla were identified (Fig. 3, Figs S3 and S4 [with standard deviation]), with members of 8 phyla representing ~95% of the total bacterial community (in order of abundance; *Actinobacteria, Proteobacteria, Cyanobacteria, Gemmatimonadetes, Chloroflexi, Bacteroidetes, Firmicutes, Armatimonadetes*). The proportion of classifiable sequences decreased substantially from phylum (98%) to genus (10%) levels, showing Namib Desert soils present numerous novel representatives of well-known edaphic phyla^41^,^61^. Nevertheless, the vast majority of sequences could be classified to class level (95% of sequences). The ubiquitous *Actinobacteria* (44.8% ± 5.6% 16S rRNA gene sequences; mean between time-points ± s.d.) and *Proteobacteria* (38.2% ± 3.6%) dominated all soil samples and also exhibited very high diversity (>300 OTUs classified to family level). This is consistent with previous phylogenetic surveys of bacterial communities in hot desert soils and soil crusts, including sites in the Negev^33^, Atacama^62^ and Namib Deserts^63^. *Cyanobacteria* comprised fewer OTUs than many of other bacterial phyla but were present at relatively high abundance (6.44% ± 1.37%; Fig. S5). The reverse was found for the *Gemmatimonadetes, Armatimonadetes, Chloroflexi* and *Bacteroidetes* phyla.

**Figure 4:**
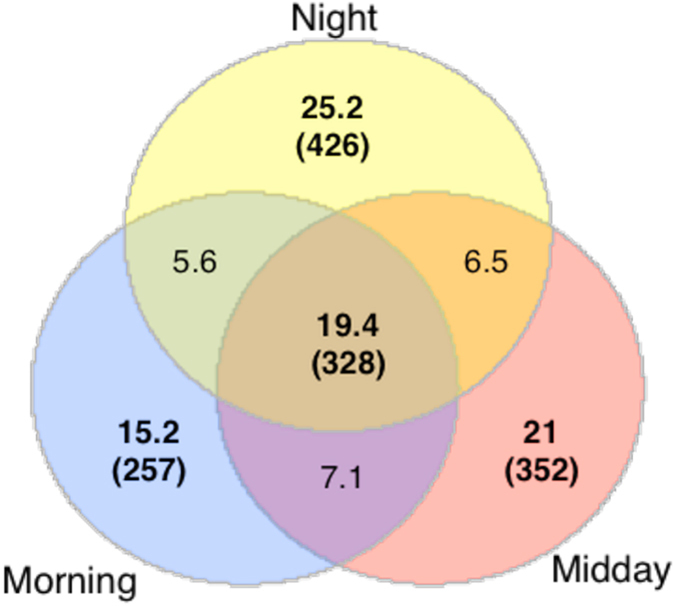
Venn diagram representing the percentage dispersal of bacterial OTUs. The data was resampled to lowest level (>14,000 sequences, 1688 OTUs0.03) between time-points. The number of reads are in parenthesis.

Strikingly, it was found that only a small fraction of the OTUs were detected at each time-point (i.e., core community - 19.4%; n = 328; Fig. 4), with the amount of OTUs recorded only at a single time-point being relatively high. For example, the night-specific OTUs represented >25% of the total bacterial activity. This pattern was also found when each day was analyzed separately (Fig. S6). PERMANOVA confirmed that the bacterial community composition exhibited significant structural shifts over each diel cycle (F_2,44_ = 1.3370, P = 0.044), supporting observations from T-RFLP analyses. We also found that the majority of the temporally-specific OTUs were members of the rare bacterial fraction (>60% of total OTUs). Indeed, the core community (<20% of total OTUs) constituted the majority of total sequence reads with a mean relative abundance value of 78% for all samples. In order to ascertain whether this rare fraction were primarily responsible for the recorded shifts in community structure, we applied the same approach with the rare fraction removed (i.e., with no singletons or doubletons), thus reducing the time-point specific OTUs from 1035 [61.4%] to 21 [4%]). A similar statistical result was recorded (Table S2), confirming that prevalent members within these hot desert soil bacterial communities also exhibited a significant shift in relative abundance patterns over a diel cycle.

### Phylogeny and distribution of diversity

Relative abundance patterns of the bacterial communities were analysed by aggregating all taxa at the phylum, class and order levels. Overall, the phyla *Actinobacteria* and *Proteobacteria* exhibited differential responses over diel cycles (Fig. 3; Fig. S3), with a strong negative correlation between their respective relative abundances (Pearson; −0.069, P < 0.01). The former exhibited a significant reduction in relative abundance from Morning to Night (ANOVA; (F_2,44_ = 5.229, P = 0.009) with a consistent pattern between days (P = 0.13). The diel patterns of sub-phylum phylogenetic groups generally reflected that at phylum level (Fig. S7). There were certain exceptions, however, with the *Micrococcales* order of the *Actinobacteria* class recording an increase from Morning to Night (Figure S7b). Within the phylum *Proteobacteria*, both β- and δ-*Proteobacteria* occurred consistently at relatively low abundance (<4% each) while γ-*Proteobacteria* showed a consistent increase in relative abundance from Morning to Night samples (Figure S7a), which was primarily attributed to the order *Pseudomonadales* (Figure S7b). Furthermore, the increased abundance of *Cyanobacteria* in Night samples could be primarily attributed to the order *Nostocales* (Figure S7b), which displayed a >6-fold increase from Morning to Night samples on day 3. The *Nostocales* group are crucial in maintaining soil N budgets through N_2_-fixation^64^, suggesting a capacity for active nitrogen cycling within this desert soil system that may be governed by temporal cues. No other phyla showed major or consistent abundance changes between time-points, although *Armatimonadetes, Bacteroidetes* and *Planctomycetes* were found to be statistically different between days (ANOVA, P < 0.05).

### Influence of abiotic factors on desert soil bacterial community structure

In order to probe the drivers of fine-scale bacterial population dynamics, we tested the possible effects of environmental factors on the bacterial community composition for all samples.

**Figure 5:**
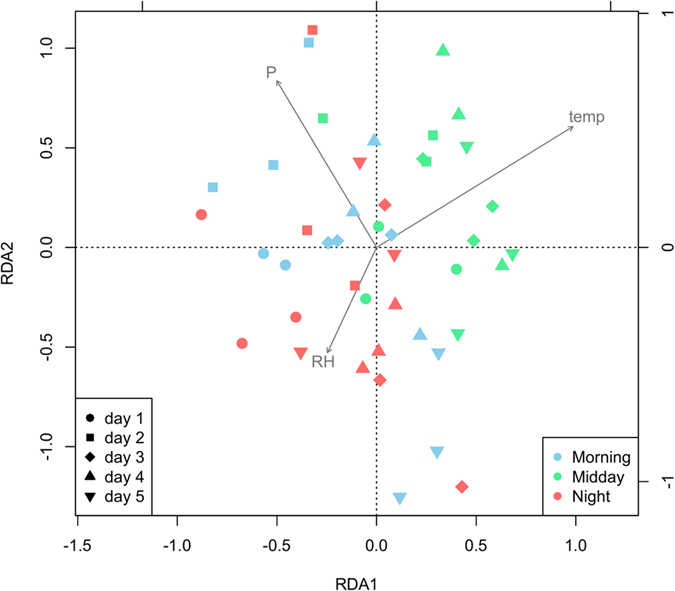
Redundancy analysis (RDA) biplot of bacterial diversity and microenvironmental parameters – Relative humidity (RH), temperature (temp) and phosphorus (P). Only the environmental variables that significantly explained variability in microbial community structure are fitted to the ordination (arrows). Each point represents a bacterial community at a specific time-point (n = 45). The direction of the arrows indicates the direction of maximum change of that variable, whereas the length of the arrow is proportional to the rate of change.

Multiple rank correlations (Spearman; global ‘BEST’ test) indicated that the abiotic parameters measured could not account satisfactorily for bacterial composition over time (P = 0.62, 99 permutations). Re-analysis of individual parameters using linear-constrained ordination redundancy analysis (RDA) with forward selection revealed that, for the physical and chemical parameters, climatic factors (temperature, F = 1.981, P = 0.02; RH, F = 1.338, P = 0.043) together with soil phosphorus content (F = 1.373, P = 0.039) had a significant influence on community composition (Fig. 5). However, these parameters only accounted for 10.5% of the total variation, suggesting that other factors were important in driving the community dynamics recorded in this study.

The extent of individual environmental variables on taxon distribution was also tested by Spearman correlations. *Actinobacteria, Acidimicrobiales, Solibrobacterales* and both β- and γ- *Proteobacteria* had strong correlations with soil temperature and %RH, while soil phosphorus content was positively correlated with the *Rhizobiales, Rhodospirillales* (α-*Proteobacteria*) and *Chroococcales (Cyanobacteria*) orders (P < 0.05). Interestingly, two orders of *Cyanobacteria*, the *Nostocales* and *Oscillatoriales*, were also positively correlated with nitrogen elements (NH_4_^+^ and NO_3_^−^) (P < 0.05). The finding that *Nostocales* group were positively correlated with nitrogen elements further enhances our view that this group may be key in driving N_2_-fixation within this desert soil system.

### Time-dependent bacterial associations revealed through co-occurrence networks

**Figure 6:**
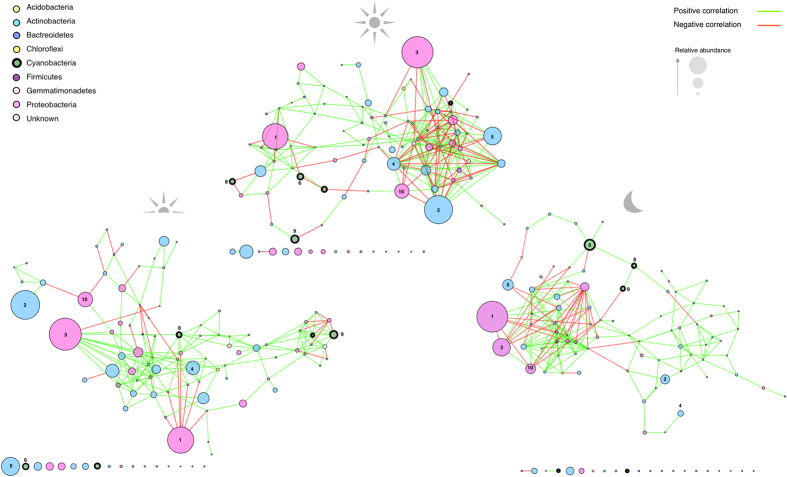
Networks (organic layout algorithm) showing the interactions between the top 100 OTUs potentially active over the diel cycle. Each node (circle) in the network symbolizes a unique OTU at the 90% threshold (family level) and the size relates to the mean relative abundance within that network directly derived from the related read counts. Each edge (connection) relates to a strong (Spearman’s ρ > 0.6) and significant (P-value < 0.01) correlation. The OTUs are numbered in accordance with overall relative abundance.

Given that the physicochemical parameters measured could not account satisfactorily for the recorded community dynamics, we investigated the possible influence of biotic associations during diel cycling. In order to do so, we generated co-occurrence networks for each time-point incorporating the five days of data (Fig. 6; organic layout algorithm). This mathematical approach has been successfully employed to infer phylogenetic and functional trait associations for edaphic and hypolithic microbial communities^33^,^51^,^53^. In the Namib Desert, this approach has previously been used to show the differences between hypolithic communities from fog- and rain- dominated zones and highlight the relevance of Cyanobacteria in potentially driving hypolithic food web compositions^51^. The networks presented in Fig. 6 comprised the most dominant fraction of the core community; i.e., the 100 most abundant and consistently detected OTUs (84% of total reads). Although present in all soil samples, this core group exhibited a reproducible non-random co-occurrence pattern against the null model at each time-point (Average C-score 63.51, P ≤ 0.001). Interestingly, the average path length (distance between all pairs of nodes), clustering and topological (tendency of nodes to share neighbors) coefficients were different for each network (Table 2), indicating that the system was interactive and dynamic (average of >5 edges [associations] per node [OTU] for each network)^53^,^65^. A level of structural homology was also evident as a primary group of nodes (module) formed a highly clustered topology at each time point; i.e., with a ‘neighborhood connectivity’ (NC, which corresponds to the number of connections between neighboring OTUs) over 7. Globally, the Midday network recorded a considerably higher NC value (6.38) when compared to the Morning (5.1) and Night networks (5.24) (Table 2). When attempting to understand the recorded patterns recorded here it is important to note that co-correlation is not necessarily indicative of biotic interactions. Indeed, the recorded patterns relating to midday community dynamics may be due to these bacterial groups responding to the variability in environmental cues (e.g., temperature) in a similar way, which would appear as co-correlation. However, modularity in community ecology can also reflect functional associations with physical contact and/or phylogenetic clustering of closely related species^66^, which has been reported to have a possible role in negating the effects of high temperatures disturbance^67^. Given the extreme temperature conditions associated with the Midday soil habitat in this system (up to 52 °C on day 2; Fig. 1), there is a likelihood that metabolic and/or functional associations could occur between adjacent temperature-tolerant bacterial taxa such as the capacity to metabolise substrates not accessible to non-thermophilic microbial communities^68^. Interestingly, *Actinobacteria* inter-phyla co-occurrence patterns (represented by OTUs with at least 2 connections) peaked at Midday (57%), the majority of which were positive (Table S3). Many of the positive associations within the primary module were between closely related radiotolerant phylotypes such as *Patulibacter* (OTU2) and *Solirubrobacter* (OTU5)^42^ (Fig. 6). This high degree of modularity incorporating similar phylotypes may partly explain the community divergence between Midday and the other time-points. Interestingly, *Proteobacteria*-affiliated OTUs exhibited the lowest level of intra-phyla co-occurrence (both positive and negative) at Midday (21%), being 2.3- and 2.2-fold lower than the Morning and Night networks, respectively, which suggest that proteobacterial taxa are more involved in multi-trophic associations during daytime habitat conditions.

The majority of *Cyanobacteria* OTUs were located externally to the primary module for each network (Fig. 6). Considering the physiological distinction between the photoautotrophic *Cyanobacteria* and the heterotrophic *Actinobacteria* and *Proteobacteria*, differences in co- occurrence profiles at different times of the diel cycle may be an indication of very fine time-scale niche partitioning^69^. Alternatively, these *Cyanobacteria* driven tertiary connections may function as inter-module hubs^51^, such as in the Midday network, where *Cyanobacteria* nodes linked the core module with a submodule that comprised a highly abundant *Proteobacteria* OTU (Fig. 6). Furthermore, a highly abundant (4% of morning reads), highly associated (7 connections; 6 positive/1 negative) *Cyanobacteria* OTU assigned to the order *Oscillatoriales* was positioned as a module hub in the Morning network, with strong inter-phyla positive co-occurrence, which is indicative of mutualistic associations. Interestingly, this module was comprised of taxonomically unique groups (distinct at class level), which may provide evidence of naturalization^70^, a concept, which suggests that co-occurring species are likely to be metabolically dissimilar so as to reduce the risk of competitive exclusion. The influence of *Cyanobacteria* OTUs as modular hubs was not recorded for the Midday and Night networks. Even low abundance phyla exhibited a high level of co-occurrence. For example, *Acidobacteria* (class *Holophagae*) only had a single representative in the core community but had 25 significant correlations (mostly positive), the majority of these being with much higher abundance OTUs in the Morning dataset (Fig. 6; Table S3). *Acidobacteria* are ubiquitous as K-strategists in edaphic niches characterized by low plant-derived carbon sources and generally more oligotrophic niches^71^,^72^. Although the functional relevance of this phylum remains unclear, the high rate of inter-phyla co-occurrence would suggest a pivotal role in edaphic processes within this hot desert soil system, with low relative abundance recorded here possibly due to an underestimation of taxa with low 16S rRNA copy numbers per genome such as *Acidobacteria*^73^.

Overall, our results clearly demonstrate that significant changes in the active fraction of Namib Desert soil bacterial communities can occur within a single day, with this study being the first to provide direct evidence of short temporal changes in undisturbed hot desert soil communities. Furthermore, we have shown that these changes are not primarily governed by abiotic factors (i.e., physicochemical variables and climatic regime) as proposed by our leading hypothesis, and that distinct microbial interactions may be important in the recorded community dynamics. The bacterial co-occurrence matrices presented here are intriguing and provide an initial glimpse of potential trait associations and mechanisms driving community organization within this system. For instance, phylogenetic clustering of desert soil bacteria can be influenced by the filtering of traits over fine-time scales, which may confer resistance to abiotic stress or competitive exclusion (e.g., high temperature resistant phylotypes; Midday function). With previous investigations focusing on refuge niche habitats as the ‘core hubs’ of microbial function and diversity in desert systems^63^,^74^,^75^,^76^,^77^, these findings are all the more striking and have implications for future desert soil research. Incorporation of additional meta-omics approaches such as metaproteomics as well as measuring photosynthetically available radiation (PAR) would be beneficial in resolving the findings recorded in this study.

### Experimental Procedures

#### Site description and sample collection

Sampling was performed between 22 and 26 April 2013 at a single undisturbed gravel-plain site (10 × 10 m quadrat; Fig. 1) adjacent to the Gobabeb Research and Training Centre, Namibia (23°33′S, 15°02′E). This region is classified as hyper-arid^8^,^37^ with low mean annual precipitation (<30 mm) and receives much of its moisture via fog events^37^.

In order to limit sampling bias and to ensure sampling homogeneity, the site was divided into a series of quadrats (x16; 2.5 × 2.5 m), which were further subdivided into four mini-quadrats (1 m^2^). Eight surface (0–5 cm) pseudo-replicate soil samples were recovered and pooled from three randomly selected (RAND()*N function in Microsoft excel) mini-quadrats at time-points reflecting the diel cycle – morning (6 am), midday (1 pm) and night (8 pm) over a 5-day period (n = 45). Each soil sample (~10 g) was supplemented with 5 ml of RNAlater (Sigma-Aldrich, Copenhagen, Denmark) and stored at −80 °C for later molecular analysis. For physicochemical analyses, ~200 g of soil was taken from each sampling zone and stored in sterile Whirlpak^®^ bags at 4 °C.

#### Climatic data and soil physicochemical analysis

Temperature (°C) and relative humidity (%RH) readings were recorded at 30 min intervals for the 5-day period using Hygrochron iButton temperature and humidity loggers (model DS1923; Embedded Data Systems, Lawrenceburg, KY, USA). These were placed at a depth of 2 cm at the center of each 2.5 m^2^ quadrat (16 in total). Physicochemical analyses were conducted at the Soil Science Laboratory of the University of Pretoria (South Africa) using standard quality control procedures^78^. Soil samples were sieved (2 mm) prior to analysis. Total organic carbon (%) was determined by a dichromate oxidation procedure^79^. A correction factor of 1.32 was used to account for incomplete oxidation of organic C^80^. Ammonium (NH_4_^+^) and nitrate (NO_3_^−^) ions were determined using steam distillation^81^. The P-bray method^82^, with Inductively Coupled Plasma-Optical Emission Spectroscopy (ICP-OES) was used to determine total organic phosphorus. The cation exchange capacity (CEC) was determined by 0.2 M ammonium acetate treatment, with subsequent concentrations of Fe, Ca, K, Mg and Na determined by ICP-OES. Soil pH was measured using a slurry technique (1:2.5 w/w soil/deionised water ratio) with a calibrated pH meter (Crison basic 20, Barcelona, Spain). The soil texture was determined by a fluid suspension method using a specialized hydrometer, based on Stokes’ law of particle dispersal^83^.

#### RNA isolation and bacterial community fingerprinting analysis

Total RNA was recovered from desert soil samples using an modified phenol-chloroform extraction technique^84^ with quantification using a Nanodrop 1000 spectrophotometer (NanoDrop Products, Wilmington, DE, USA). RNA preparations were treated with RQ1 RNase-Free (Promega) to remove contaminating DNA, with subsequent purification (RNeasy; Qiagen, GmbH, Hilden, Germany) and reverse transcription (Supersript^®^ III first strand synthesis; Invitrogen). Bacterial T-RFLP analysis of the cDNA products was done using 16S rRNA gene PCR primers 341 F (5′-FAM-CCTACGGGAGGCAGCAG-3′) and 908 R (5′-CCGTCAATTCCTTTRAGTTT-3′)^84^. Thermal cycling conditions were 94 °C for 5 min; 30 cycles of 94 °C for 30S, 55 °C for 20 s and 72 °C for 105 s; 72 °C for 10 min. Each 50 μl T-RFLP reaction comprised 1X DreamTaqTM reaction buffer (Fermentas, USA), 0.1 mM dNTPs, 20 mg.ml^−1^ bovine serum albumin, 0.5 μM of each primer, 2 U of DreamTaqTM polymerase (Fermentas, USA) and 10 ng of template DNA or cDNA. The resulting PCR products were purified using the NucleoSpin^®^ Kit (Macherey-Nagel, Gmbh & Co. KG, Düren, Germany), with 200 ng digested (normalized spectrophotometrically; Nanodrop 1000) using 1 U HaeIII in 2× restriction enzyme buffer (Fermentas, USA) at 37 °C overnight with an additional purification step using the NucleoSpin^®^ Kit. Separation of restriction fragments was achieved by capillary electrophoresis using an ABI 3130XL Genetic Analyzer (Applied Biosystems, Foster City, CA, USA) with the LIZ^®^600 Size Standard. The resulting fragment profiles were exported and analyzed using Peak Scanner 1.0 (Applied Biosystems) with the default 5RFU cutoff. In order to eliminate any inaccuracies in the data, true peaks and fragments of similar size were identified and binned using the software R and Perl^85^, Fragment lengths within 1 bp of each other were considered to be the same operational taxonomic unit (OTU) and peaks within 3 standard deviation of the noise baseline were removed. A matrix of the T-RFLP community fingerprint patterns (‘ClusBinMatrix’) for the relative abundance (area under the peak) of individual OTUs for each soil sample was generated for subsequent analysis.

#### 454 Pyrosequencing of PCR amplicons

Bacterial community composition was estimated by bacterial tag-encoded FLX amplicon pyrosequencing (bTE-FAP) using the GS FLX titanium-sequencing platform according to MR DNA protocols (www.mrdnalab.com). Briefly, PCR amplicons spanning the hypervariable regions V1-V3 of the bacterial 16S rRNA gene were generated from each cDNA sample (n = 45) using the primer pair 27 F/519R^86^, with unique multiplex identification tags (error corrected MIDs or ‘barcodes’) added to the primers for each sample. A single-step 30 cycle PCR using HotStarTaq Plus Master Mix Kit (Qiagen, Valencia, CA) was used under the following conditions: 94 °C for 3 minutes, 28 cycles of 94 °C for 30 seconds; 53 °C for 40 seconds and 72 °C for 1 minute; followed by an final elongation step at 72 °C for 5 minutes. The PCR amplicons were then purified using Agencourt Ampure beads (Agencourt Bioscience Corporation, MA, USA) and sequenced utilizing a Roche 454 FLX Titanium NG sequencing platform (Molecular Research LP, Shallowater, Texas). The pyrosequencing flograms were derived from the original SFF files and analyzed using a pre-established pipeline in MOTHUR (version 1.33.3)^87^. A stringent approach to this analysis was used, with no primer and barcode sequence mismatches permitted. Errors in sequencing were reduced by the trim.flows and shhh.flows commands, the latter incorporating an implementation of PyroNoise^88^ that uses the expectation-maximization algorithm. Sequences with ambiguities and/or shorter than 200 bps were removed. After configuring the data to comprise unique sequences, further de-noising was done using the ‘pre.cluster’ command, which employs a pseudo-single linkage algorithm to remove sequences likely due to pyrosequencing errors^89^.

Finally, the sequences were checked for PCR chimeras using the chimera.slayer command^90^. Aligned sequences were used to construct distance matrices and to cluster the sequences into operational taxonomic units (OTUs) at the 97% (species level) and 90% (family level) cutoffs in MOTHUR. Bacterial sequences were classified using CREST^91^ with the Silvamod database and MEGAN with LCA parameters min bit score 250, top percent 2 (50 best blast hits). Rank abundance data were generated for all samples with rarefaction curves and other population diversity indices (for example, Chao1, Shannon evenness) calculated in MOTHUR after we rarefied the species richness to the same number of individuals to ensure sequence consistency between samples^92^. Venn diagrams for days and time-points were generated using the R statistical package (v.2.14.0)^93^.

The SFF file containing the sequence data is available at the NCBI Sequence Read Archive under the accession number PRJNA253979.

### Data analysis

All statistical analyses were carried out using R, including functions from the vegan package (version 2.0-9). For T-RFLP and pyrosequencing analysis, the dependent variables, day (i.e., day 1, day 2, day 3, day 4 and day 5) and time-point (i.e., morning, midday and night) were treated as fixed factors. QQ-plots and frequency histograms indicated that residuals did not meet the assumptions required for parametric tests^94^. Therefore, independent variables (x) (i.e., all soil parameters and all community data) were transformed according to ln (x + 1).

Linear models were used to determine significance between both days and time-points for the different soil parameters (lm function). T-RFLP and pyrosequencing community profiles (employing Hellinger transformation) were visualized using non-metric multidimensional scaling (nMDS) analyses (meta.mds function) using the Bray-Curtis dissimilarity metrics. In order to test for significant differences in bacterial composition between samples, permutational multivariate analysis of variance (PERMANOVA) was used with either day (day 1, day 2, day 3, day 4, day 5) or time-point (morning/midday/night) used as factors (Adonis function). We also tested for an interaction effect between days and time-points (model; day*time). In order to determine which experimental groups were distinguishable, *post hoc* Tukey tests were used for pairwise comparisons. In order to test the influence of abiotic factors on variations within bacterial community structure, Biota Environment STepwise matching (global ‘BEST’ test) in the Primer6 statistical package (Primer-E Ltd, UK)^95^ was undertaken. The soil physicochemical data was standardized. Spearman rank correlations between the abiotic variables and the bacterial communities were then calculated over the sampling period (99 permutations). Redundancy analysis (RDA; rda function) with forward selection was used to evaluate the effects of the environment on community composition.

Co-occurrence networks were generated comprising consistently detected and highly abundant OTUs; i.e., the ‘core’ community. Non-random co-occurrence patterns were first tested with the checkerboard score (C-score) under the null hypothesis of random community assembly^96^, where 5,000 matrices were randomly generated from the 16SrRNA gene data (oecosimu function with nestedchecker in bipartite package). All possible Spearman rank correlations between OTUs were then calculated with stringent parameters set (interaction between OTUs valid if >0.6 value for Spearman’s correlation coefficient [ρ] and P-value < 0.01) as previously described^53^. A graphical representation of the filtered dataset was prepared using Cytoscape^97^. The nodes in the constructed network represented OTUs with the edges (connections) indicating significant inter-nodal correlations. Topological coefficients of the resulting networks were then calculated in order to derive comparisons, which included average node connectivity, average path lengths, cumulative degree distribution and clustering coefficients.

## Additional Information

**How to cite this article**: Gunnigle, E. *et al*. Diel-scale temporal dynamics recorded for bacterial groups in Namib Desert soil. *Sci. Rep.* **7**, 40189; doi: 10.1038/srep40189 (2017).

**Publisher's note:** Springer Nature remains neutral with regard to jurisdictional claims in published maps and institutional affiliations.

## Supplementary Material

Supplementary Information

## Figures and Tables

**Table 1:** Sampling data, distribution and diversity of OTUs (97% cutoff, subsampled).

Day	Time-point	Richness			Diversity		Evenness
		Observed	Chao1	ACE	Shannon	Inv. Simpson	Shannon even
1	Morning	287(41)	205(64)	253(110)	4.4(0.3)	64.5(30.4)	0.9(0.02)
	Midday	435(37)	337(292)	556(292)	4.3(0.7)	63.2(15.6)	0.88(0.06)
	Night	532(84)	382(55)	609(298)	4.7(0.3)	69.9(49.6)	0.89(0.06)
2	Morning	337(39)	189(101)	251(173)	4.1(0.7)	47.5(27.9)	0.87(0.06)
	Midday	341(37)	194(85)	278(164)	4.2(0.4)	44.9(19.9)	0.88(0.03)
	Night	385(40)	283(169)	299(189)	4.1(0.8)	47.1(37.6)	0.84(0.08)
3	Morning	490(14)	490(208)	923(299)	4.6(0.4)	80(47.8)	0.91(0.03)
	Midday	399(20)	232(48)	234(48)	4.4(0.3)	51.5(9.6)	0.9(0.02)
	Night	425(44)	300(52)	297(76)	4.2(0.6)	27.9(17.1)	0.88(0.08)
4	Morning	463(18)	336(223)	492(92)	4.5(0.6)	97.3(45.8)	0.9(0.05)
	Midday	507(74)	414(162)	561(248)	4.6(0.3)	64.4(38.2)	0.93(0.02)
	Night	242(40)	129(108)	180(109)	3.2(0.9)	24.4(12.4)	0.74(0.09)
5	Morning	300(88)	154(86)	167(81)	3.8(0.7)	30.7(28.4)	0.83(0.08)
	Midday	419(41)	291(161)	422(189)	4.3(0.7)	48.5(33.6)	0.87(0.07)
	Night	389(23)	224(27)	325(98)	4.4(0.1)	53.6(13.5)	0.9(0.02)

Data shown is derived from 3 individual biological replicates for each time-point except Day 1 Morning where as a consequence of sequencing error, only two biological replicates were used.

Standard deviation in parenthesis.

**Table 2:** Network parameters for morning, midday and night bacterial communities.

	Total number of correlations (+/−)	Neighbourhood connectivity^a^	Shortest path length^b^	Clustering coefficient (*C*_n_)^c^	Topological coefficient^d^
Morning	375 (328/47)	5.1	3.41	0.28	0.31
Midday	488 (297/191)	6.38	3.23	0.26	0.3
Night	393 (313/80)	5.24	3.25	0.25	0.28

^a^Connectivity rate between OTUs (edges).

^b^Length of shortest path between all OTUs.

^c^Ratio of *N/M*, where *N* is number of connections between neighbors of each OTU, and *M* is the maximum number of connections possible between the neighbors of each OTU.

^d^Relative measurement for level to which each OTU shared neighbors with other OTUs.

^a–d^Each value is the mean of all OTUs_0.1_ (n = 100) analysed.

## References

[b1] Thibault K. M. & Brown J. H. Impact of an extreme climatic event on community assembly. Proc. Natl. Acad. Sci. USA 109, 3410–3415 (2008). 10.1073/pnas.0712282105 PMC2265133 18303115

[b2] Lavergne S., Mouquet N., Thuiller W. & Ronce O. Biodiversity and climate change: Integrating evolutionary and ecological responses of species and communities. Ecol. Evol. Sys. 4, 321–50 (2010).

[b3] Nørgaard T. & Dacke M. Fog-basking behaviour and water collection efficiency in Namib Desert Darkling beetles. Front. Zool. 7, 23 (2010). 20637085 10.1186/1742-9994-7-23 PMC2918599

[b4] Eppel A., Shaked R., Eshel G., Barek S. & Rachmilevitch S. Low induction of non-photochemical quenching and high photochemical efficiency in the annual desert plant *Anastatica hierochuntica*. Phys. Plantarum 151, 544–558 (2014). 10.1111/ppl.12146 24372077

[b5] Ma T. . Genomic insights into salt adaptation in a desert poplar. Nat Comm. 4, 2797 (2014). 10.1038/ncomms3797 24256998

[b6] Andrew D. R. . Abiotic factors shape microbial diversity in Sonoran Desert soils. Appl. Environ. Microbiol. 78, 7527–7537 (2012). 22885757 10.1128/AEM.01459-12 PMC3485727

[b7] Pointing B. & Belnap J. Microbial colonization and controls in dryland systems. Nat. Rev. 10, 551–562 (2012). 10.1038/nrmicro2831 22772903

[b8] Makhalanyane T. P. . Microbial ecology of hot desert edaphic systems. FEMS Microbiol. Rev. 39, 203–221 (2015). 25725013 10.1093/femsre/fuu011

[b9] IPCC, Climate Change 2013. The Physical Science Basis. Contribution of Working Group I to the Fifth Assessment Report of the Intergovernmental Panel on Climate Change (Cambridge University Press, 2013).

[b10] Bell C. W. . Soil microbial and nutrient responses to 7 years of seasonally altered precipitation in a Chihuahuan Desert grassland. Glob. Change. Biol. 20, 1657–1673 (2014). 10.1111/gcb.12418 24115607

[b11] Cheng X. . Summer rain pulse size and rainwater uptake by three dominant desert plants in a desertified grassland ecosystem in northwestern China. Plant. Ecol. 184, 1–12 (2006).

[b12] Nano C. E. M. & Pavey C. R. Refining the ‘pulse-reserve’ model for arid central Australia, Seasonal rainfall, soil moisture and plant productivity in sand ridge and stony plain habitats of the Simpson Desert. Aust. Ecol. 38, 741–753 (2013).

[b13] Dong X., Grimm N. B., Ogle K. & Franklin J. Temporal variability in hydrology modifies the influence of geomorphology on wetland distribution along a desert stream. J. Ecol. 104, 31–32 (2015).

[b14] Bowker M. A., Reed S. C., Belnap S. L. & Phillips S. L. Temporal variation in community composition, pigmentation, and Fv/Fm of desert cyanobacterial soil crusts. Micro. Ecol. 43, 13–25 (2002). 10.1007/s00248-001-1013-9 11984625

[b15] Clark J. S., Campbell J. H., Grizzle H., Acosta-Martinez V. & Zak J. C. Soil microbial community response to drought and precipitation variability in the Chihuahuan Desert. Micro. Ecol. 57, 248–260 (2009). 10.1007/s00248-008-9475-7 19067031

[b16] Pasternak Z. . Spatial and temporal biogeography of soil microbial communities in arid and semiarid regions. PloS One. 8, e69705 (2013). 23922779 10.1371/journal.pone.0069705 PMC3724898

[b17] Saul-Tcherkas V., Unc A. & Steinberger Y. Soil microbial diversity in the vicinity of desert shrubs. Micro. Ecol. 65, 689–699 (2013). 10.1007/s00248-012-0141-8 23192699

[b18] Manzoni S., Schimel J. P. & Porporato A. Responses of soil microbial communities to water stress, results from a meta-analysis. Ecology. 93, 930–938 (2012). 22690643 10.1890/11-0026.1

[b19] Frossard A., Gerull L., Mutz M. & Gessner M. O. Disconnect of microbial structure and function, enzyme activities and bacterial communities in nascent stream corridors. ISME J. 6, 680–691 (2012). 22030674 10.1038/ismej.2011.134 PMC3280134

[b20] Nielsen U. N. & Ball B. A. Impacts of altered precipitation regimes on soil communities and biogeochemistry in arid and semi arid ecosystems. Glob. Change Biol. 21, 1407–1421 (2015). 10.1111/gcb.12789 25363193

[b21] Fierer N. & Jackson R. B. The diversity and biogeography of soil bacterial communities. Proc. Natl. Acad. Sci. USA 103, 626–631 (2006). 16407148 10.1073/pnas.0507535103 PMC1334650

[b22] Wang J. . Phylogenetic beta diversity in bacterial assemblages across ecosystems, deterministic versus stochastic processes. ISME J. 7, 1310–1321 (2013). 23446837 10.1038/ismej.2013.30 PMC3695296

[b23] Rajeev L. . Dynamic cyanobacterial response to hydration and dehydration in a desert biological soil crust. ISME J. 7, 2178–2191 (2013). 23739051 10.1038/ismej.2013.83 PMC3806265

[b24] Wang W., Wang Y., Shu X. & Zhang Q. Physiological responses of soil crust-forming Cyanobacteria to diurnal temperature variation. J. Bas. Micro. 53, 72–80 (2013). 10.1002/jobm.201100510 22581520

[b25] Zhang N., Xia J., Yu X., Ma K. & Wan S. Soil microbial community changes and their linkages with ecosystem carbon exchange under asymmetrically diurnal warming. Soil. Biol. Biochem. 43, 2053–2059 (2011).

[b26] Ma J., Wang Z.-Y., Stevenson B. A., Zheng X.-J. & Li Y. An inorganic CO_2_ diffusion and dissolution process explains negative CO_2_ fluxes in saline/alkaline soils. Sci. Rep. 3, 2025 (2013). 23778238 10.1038/srep02025 PMC3685845

[b27] Feng W. . Impact of environmental factors and biological soil crust types on soil respiration in a desert ecosystem. PloS One 9, e102954 (2014). 25050837 10.1371/journal.pone.0102954 PMC4106843

[b28] Wang B. . Soil moisture modifies the response of soil respiration to temperature in a desert shrub ecosystem. Biogeosciences 11, 259–268 (2014).

[b29] Beier C. . Carbon and nitrogen cycles in European ecosystems respond differently to global warming. Sci. Tot. Environ. 407, 692–697 (2008). 10.1016/j.scitotenv.2008.10.001 18930514

[b30] Xia J. Y., Niu S. L. & Wan S. Q. Response of ecosystem carbon exchange to warming and nitrogen addition during two climatologically contrasting growing seasons in a temperate steppe. Glob. Chan. Biol. 15, 1544–1556 (2009).

[b31] Su H., Feng J., Axmacher J. C. & Sang W. Asymmetric warming significantly affects net primary production, but not ecosystem carbon balances of forest and grassland ecosystems in northern China. Sci. Rep. 5, 9115 (2015). 25766381 10.1038/srep09115 PMC4357852

[b32] Noy-Meir I. Desert ecosystems. I. Environment and producers. Ann. Rev. Ecol. Sys. 4, 25–52 (1973).

[b33] Angel R. & Conrad R. Elucidating the microbial resuscitation cascade in biological soil crusts following a simulated rain event. Environ. Microbiol. 15, 2799–2815 (2013). 23648088 10.1111/1462-2920.12140

[b34] Belnap J., Welter J. R., Grimm N. B., Barger N. & Ludwigm J. A. Linkages between microbial and hydrological processes in arid and semiarid watersheds. Ecology 86, 298–307 (2005).

[b35] Collins S. L. . Pulse dynamics and microbial processes in arid land ecosystems. J. Ecol. 96, 413–420 (2008).

[b36] Frossard A., Ramond J.-B., Seely M. & Cowan D. A. Water regime history drives responses of soil Namib Desert microbial communities to wetting events. Sci. Rep. 5, 12263 (2015). 26195343 10.1038/srep12263 PMC4508562

[b37] Eckardt F. D. . The nature of moisture at Gobabeb, in the central Namib Desert. J. Arid. Environ. 93, 7–19 (2013).

[b38] Crits-Christoph A. . Colonization patterns of soil microbial communities in the Atacama Desert. Microbiome 1, 28 (2013). 24451153 10.1186/2049-2618-1-28 PMC3971613

[b39] Smith J. J., Tow L. A., Stafford W., Cary C. & Cowan D. A. Bacterial diversity in three different Antarctic cold desert mineral soils. Micro. Ecol. 51, 413–421 (2006). 10.1007/s00248-006-9022-3 16596438

[b40] Lee C. K., Barbier B. A., Bottos E. M., McDonald I. R. & Cary S. C. The Inter-Valley Soil Comparative Survey, the ecology of Dry Valley edaphic microbial communities. ISME J. 6, 1046–1057 (2012). 22170424 10.1038/ismej.2011.170 PMC3329106

[b41] Fierer N. . Cross-biome metagenomic analyses of soil microbial communities and their functional attributes. Proc. Natl. Acad. Sci. USA 109, 21390–21395 (2012). 23236140 10.1073/pnas.1215210110 PMC3535587

[b42] Neilson J. W. . Life at the hyperarid margin, novel bacterial diversity in arid soils of the Atacama Desert, Chile. Extremophiles 16, 553–566 (2012). 22527047 10.1007/s00792-012-0454-z

[b43] Fernandez-Aunión C. . Biosynthesis of compatible solutes in rhizobial strains isolated from Phaseolus vulgaris nodules in Tunisian fields. BMC Microbiol. 10, 192 (2010). 20633304 10.1186/1471-2180-10-192 PMC2918589

[b44] LeBlanc J. C., Gonçalves E. R. & Mohn W. W. Global response to desiccation stress in the soil *Actinomycete Rhodococcus jostii* RHA1. App. Environ. Microbiol. 74, 2627–2636 (2008). 10.1128/AEM.02711-07 PMC2394902 18326668

[b45] Seely M. K. & Hamilton W. J. Fog catchment sand trenches constructed by tenebrionid beetles, *Lepidochora*, from the Namib Desert. Science 193, 484–486 (1976). 17841821 10.1126/science.193.4252.484

[b46] Ebner M. E. M., Miranda T. & Roth-Nebelsick A. Efficient fog harvesting by *Stipagrostis sabulicola* (Namib dune bushman grass). J. Arid. Environ. 75, 524–531 (2011).

[b47] Warren-Rhodes K. A. . Hypolithic Cyanobacteria, Dry Limit of Photosynthesis, and Microbial Ecology in the Hyperarid Atacama Desert. Micro. Ecol. 52, 389–398 (2006). 10.1007/s00248-006-9055-7 16865610

[b48] Azúa-Bustos A. . Hypolithic *Cyanobacteria* supported mainly by fog in the coastal range of the Atacama Desert. Microb. Ecol. 61, 568–581 (2011). 21188376 10.1007/s00248-010-9784-5

[b49] Stomeo F. . Hypolithic and soil microbial community assembly along an aridity gradient in the Namib Desert. Extremophiles 17, 329–337 (2013). 23397517 10.1007/s00792-013-0519-7

[b50] Warren-Rhodes K. A. . Physical ecology of hypolithic communities in the central Namib Desert, The role of fog, rain, rock habitat, and light. J. Geophys. Res. 118, 1451–1460 (2013).

[b51] Valverde A., Makhalanyane T. P., Seely M. & Cowan D. A. Cyanobacteria drive community composition and functionality in rock–soil interface communities. Mol. Ecol. 24, 812–821 (2015). 25640844 10.1111/mec.13068

[b52] Cáceres L. . Relative humidity patterns and fog water precipitation in the Atacama Desert and biological implications. J. Geophy. Res. 112, G4 (2007).

[b53] Barberán A., Bates S. T., Casamayor E. O. & Fierer N. Using network analysis to explore co-occurrence patterns in soil microbial communities. ISME J. 6, 343–351 (2012). 21900968 10.1038/ismej.2011.119 PMC3260507

[b54] Tobback J., Boerjan B., Vandersmissen H. P. & Huybrechts R. The circadian clock genes affect reproductive capacity in the desert locust *Schistocerca gregaria*. Insect Biochem. Mol. Biol. 41, 313–321 (2011). 21295143 10.1016/j.ibmb.2011.01.008

[b55] Walsberg G. Small mammals in hot deserts, some generalizations revisited. Biosci. 50, 109–120 (2000).

[b56] Nilsen E. T., Sharifi M. R., Rundel P. W., Jarrell W. M. & Virginia R. A. Diurnal and seasonal water relations of the desert phreatophyte *Prosopis glanulosa* (Honey Mesquite) in the Sonoran Desert of California. Ecology 64, 1381–1393 (1983).

[b57] Dvornyk V. & Jahan A. S. Extreme conservation and non-neutral evolution of the *cpm*A circadian locus in a globally distributed *Chroococcidiopsis* sp. from naturally stressful habitats. Mol. Biol. Evol. 29, 3899–3907 (2012). 22844070 10.1093/molbev/mss191 PMC3697812

[b58] Johnson C. H. Testing the adaptive value of circadian systems. Meth. Enzymol. 393, 818–837 (2005). 15817326 10.1016/S0076-6879(05)93043-7

[b59] Saikaly P. E., Stroot P. G. & Oether D. B. Use of 16S rRNA gene terminal restriction fragment analysis to assess the impact of solids retention time on the bacterial diversity of activated sludge. Appl. Environ. Micro. 71, 5814–5822 (2005). 10.1128/AEM.71.10.5814-5822.2005 PMC1265999 16204492

[b60] Lynch M. D. J. & Neufeld J. D. Ecology and exploration of the rare biosphere. Nat. Rev. Microbiol. 13, 217–229 (2015). 25730701 10.1038/nrmicro3400

[b61] Janssen P. H. Identifying the Dominant Soil Bacterial Taxa in Libraries of 16S rRNA and 16S rRNA Genes. Appl. Environ. Microbiol. 72, 1719–1728 (2006). 16517615 10.1128/AEM.72.3.1719-1728.2006 PMC1393246

[b62] Connon S. A., Lester E. D., Shafaat H. S., Obenhuber D. C. & Ponce A. Bacterial diversity in hyperarid Atacama Desert soils. J. Geophy. Res. 112, G4 (2007).

[b63] Makhalanyane T. P. . Evidence of species recruitment and development of hot desert hypolithic communities. Environ. Microbiol. Rep. 5, 219–224 (2013). 23584965 10.1111/1758-2229.12003

[b64] Boison G., Mergel A., Jolkver H. & Bothe H. Bacterial life and dinitrogen fixation at a gypsum rock. Appl. Environ. Microbiol. 70, 7070–7077 (2004). 15574902 10.1128/AEM.70.12.7070-7077.2004 PMC535155

[b65] Kara E. L., Hanson P. C., Hu Y. H., Winslow L. & McMahon K. D. A decade of seasonal dynamics and co-occurrences within freshwater bacterioplankton communities from eutrophic Lake Mendota, WI, USA. ISME J. 7, 680–684 (2013). 23051691 10.1038/ismej.2012.118 PMC3578560

[b66] Olesen J. M., Bascompte J., Dupont Y. L. & Jordano P. The modularity of pollination networks. Proc. Natl. Acad. Sci. USA 104, 19891–19896 (2007). 18056808 10.1073/pnas.0706375104 PMC2148393

[b67] Ding J. . Integrated metagenomics and network analysis of soil microbial community of the forest timberline. Sci. Rep. 5, 7994 (2015). 25613225 10.1038/srep07994 PMC4303876

[b68] Cong J. . Analyses of soil microbial community compositions and functional genes reveal potential consequences of natural forest succession. Sci. Rep. 5, 10007 (2015). 25943705 10.1038/srep10007 PMC4421864

[b69] Caruso T. . Stochastic and deterministic processes interact in the assembly of desert microbial communities on a global scale. ISME J. 5, 1406–1413 (2011). 21368908 10.1038/ismej.2011.21 PMC3160679

[b70] Zelezniak A. . Metabolic dependencies drive species co-occurrence in diverse microbial communities. Proc. Natl. Acad. Sci. USA 112, 6449–6454 (2015). 25941371 10.1073/pnas.1421834112 PMC4443341

[b71] Fierer N. . Metagenomic and small-subunit rRNA analyses reveal the genetic diversity of bacteria, archaea, fungi, and viruses in soil. Proc. Natl. Acad. Sci. USA 73, 7059–7066 (2007). 10.1128/AEM.00358-07 PMC2074941 17827313

[b72] Naether A. . Environmental factors affect Acidobacterial communities below the subgroup level in grassland and forest soils. Appl. Environ. Microbiol. 78, 7398–7406 (2012). 22885760 10.1128/AEM.01325-12 PMC3457104

[b73] Vétrovský T. & Baldrian P. The variability of the 16S rRNA gene in bacterial genomes and it’s consequences for bacterial community analyses. PloS One 8, e57923 (2013). 23460914 10.1371/journal.pone.0057923 PMC3583900

[b74] Chan Y., Van Nostrand J. D., Zhou J., Pointing S. B. & Farrell R. L. Functional ecology of an Antarctic Dry Valley. Proc. Natl. Acad. Sci. USA 110, 8990–8995 (2013). 23671121 10.1073/pnas.1300643110 PMC3670347

[b75] Garcia-Pichel F., Lopez-Cortez A. & Nubel U. Phylogenetic and morphological diversity of *Cyanobacteria* in soil desert crusts from the Colorado Plateau. Appl. Environ. Microbiol. 67, 1902–1910 (2001). 11282648 10.1128/AEM.67.4.1902-1910.2001 PMC92812

[b76] Nagy M. L., Perez A. & Garcia-Pichel F. The prokaryotic diversity of biological soil crusts in the Sonoran Desert. FEMS Microbiol. Ecol. 54, 233–245 (2005). 16332322 10.1016/j.femsec.2005.03.011

[b77] Lacap D. C., Warren-Rhodes K. A., McKay C. P. & Pointing S. B. *Cyanobacteria* and chloroflexi-dominated hypolithic colonization of quartz at the hyper-arid core of the Atacama Desert, Chile. Extremophiles 15, 1–38 (2011). 21069402 10.1007/s00792-010-0334-3 PMC3017302

[b78] SSSA. Methods of soil analysis, Part 3. *Soil Sci. Soc. America* (1996).

[b79] Nelson D. W. & Sommers L. E. Total carbon, organic carbon and organic matter. In Methods of soil analysis. Part 2 570–571. Am. Soc. Agron. (1982).

[b80] Liu Z., Shao M. & Wang Y. Effect of environmental factors on regional soil organic carbon stocks across the Loess Plateau region, China. Agri. Ecosys. Environ. 142, 184–194 (2011).

[b81] Keeney D. R. & Nelson D. W. Nitrogen in organic forms. Pages 643–698 In Page A. L. . Eds. Methods of soil analysis. Part 2. Agronomy No. 9, Amer. Soc. Agron. (1982).

[b82] Bray R. H. & Kurtz L. T. Determination of total, organic, and available forms of phosphorus in soils. Soil. Sci. 59, 39–45 (1945).

[b83] Tromp-van Meerveld H. J. . Influence of sediment settling velocity on mechanistic soil erosion modeling. Wat. Resour. Res. 44, 6 (2008).

[b84] Gunnigle E., Ramond J.-B., Frossard A., Seely, & Cowan, A sequential co-extraction method for DNA, RNA and protein recovery from soil for future system-based approaches. J. Microbiol. Meth. 103, 118–123 (2014). 10.1016/j.mimet.2014.06.004 24929037

[b85] Abdo Z. . Statistical methods for characterizing diversity of microbial communities by analysis of terminal restriction fragment length polymorphisms of 16S rRNA genes. Environ. Microbiol. 8, 929–938 (2006). 16623749 10.1111/j.1462-2920.2005.00959.x

[b86] Mao D. P., Zhou Q., Chen C. Y. & Quan Z. X. Coverage evaluation of universal bacterial primers using the metagenomic datasets. BMC Microbiol. 12, 66 (2012). 22554309 10.1186/1471-2180-12-66 PMC3445835

[b87] Schloss P. D. . Introducing mothur, open-source, platform-independent, community-supported software for describing and comparing microbial communities. Appl. Environ. Micro. 75, 7537–7537 (2009). 10.1128/AEM.01541-09 PMC2786419 19801464

[b88] Quince C., Lanzen A., Davenport R. J. & Turnbaugh P. J. Removing noise from pyrosequenced amplicons. BMC Bioinform. 12, 38 (2011). 10.1186/1471-2105-12-38 PMC3045300 21276213

[b89] Huse S. M., Welch D. M., Morrison H. G. & Sogin M. L. Ironing out the wrinkles in the rare biosphere through improved OTU clustering. Environ. Microbiol. 12, 1889–1898 (2010). 20236171 10.1111/j.1462-2920.2010.02193.x PMC2909393

[b90] Haas B. J. . Chimeric 16S rRNA sequence formation and detection in Sanger and 454-pyrosequenced PCR amplicons. Gen. Res. 21, 494–504 (2011). 10.1101/gr.112730.110 PMC3044863 21212162

[b91] Lanzén A. . CREST–Classification resources for environmental sequence tags. PloS ONE 7, e4933 (2012). 10.1371/journal.pone.0049334 PMC3493522 23145153

[b92] Bengtsson M. M., Sjøtun K., Lanzén A. & Øvreås L. Bacterial diversity in relation to secondary production and succession on surfaces of the kelp Laminaria hyperborean. ISME J. 6, 2188–2198 (2012). 22763650 10.1038/ismej.2012.67 PMC3505018

[b93] R. Core Team. R, a language and environment for statistical computing. Vienna, Austria, R Foundation for Statistical Computing, Available from: http://www.R-project.org/ (2012).

[b94] Frossard A., Gerull L., Mutz M. & Gessner M. O. Disconnect of microbial structure and function: enzyme activities and bacterial communities in nascent stream corridors. ISME J. 6, 680–691 (2012). 22030674 10.1038/ismej.2011.134 PMC3280134

[b95] Clarke K. R. & Gorley R. N. PRIMER v6: user manual. Plymouth Marine Laboratory (2006).

[b96] Caruso T. . Stochastic and deterministic processes interact in the assembly of desert microbial communities on a global scale. ISME J. 5, 1406–1413 (2011). 21368908 10.1038/ismej.2011.21 PMC3160679

[b97] Shannon P. . Cytoscape: A software environment for integrated models of biomolecular interaction networks. Gen. Res. 13, 2498–2504 (2003). 10.1101/gr.1239303 PMC403769 14597658

