# Supplementary material for: Diel-scale temporal dynamics recorded for bacterial groups in Namib Desert soil
Source: Sci Rep. 2017 Jan 10;7:40189. doi: 10.1038/srep40189 (PMC5223211; doi:10.1038/srep40189)
Supplement: Supplementary Information [file srep40189-s1.pdf]

Title page

Supplementary data

Title: Diel-scale temporal dynamics recorded for bacterial groups in Namib Desert soil

Authors: Eoin Gunnigle (EG)[1], Aline Frossard (AF)[1], Jean-Baptiste Ramond (J-BR)[1], Leandro Guerrero (LG)[1], Mary Seely (MS) [2,3] and Don A Cowan (DAC)[1]

[1] Centre for Microbial Ecology and Genomics, Genomic Research Institute, Department of Genetics, University of Pretoria, South Africa.

[2] Gobabeb Research and Training Centre, Walvis Bay, Namibia.

[3] School of Animal, Plant and Environmental Sciences University of the Witwatersrand, South Africa.

Key words: Hot desert, Namib, bacteria, soil, diel, pyrosequencing

Correspondence:

Jean-Baptiste Ramond

Centre for Microbial Ecology and Genomics, Natural Science II, Office 3-20, University of Pretoria, Hatfield 0028, Pretoria, South Africa.

Phone: +27 (0) 12 420 6980 Email: jean-baptiste.ramond@up.ac.za

Running title: Dynamic bacterial communities in hot desert soil

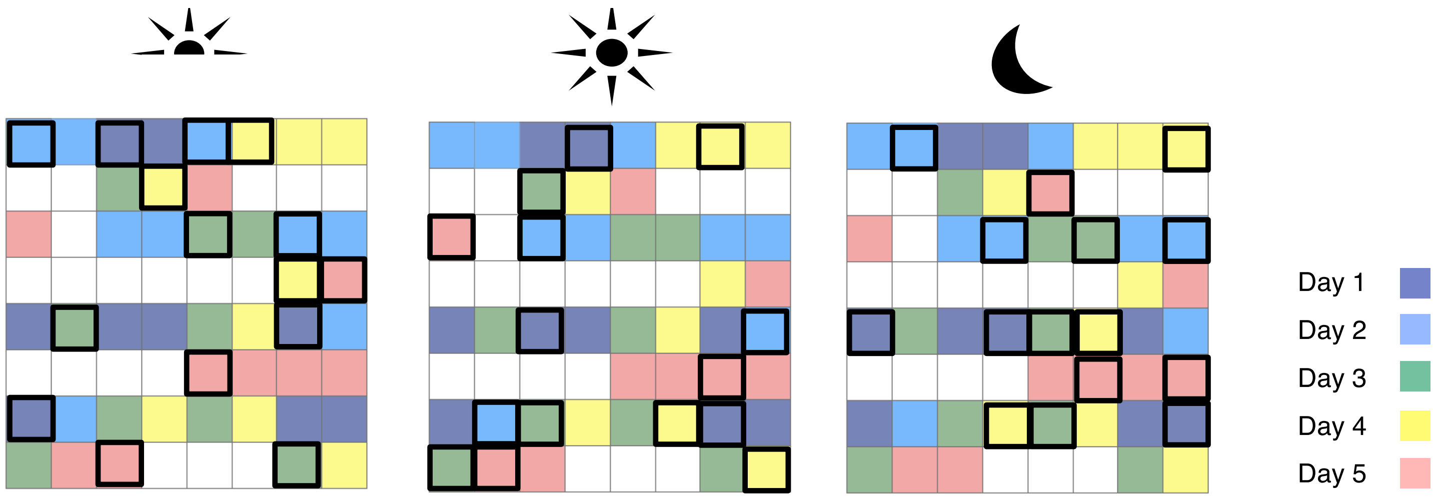

pH

7.2	6.8	7.2	6.3	6.7	8	5.9	6.8
		6.5	7	6.3			
7.4		6.6	7.4	7	8.2	7.6	6.7
						8.9	7.1
7	7.8	7.2	7.5	7.1	7.3	7.4	6.6
				7.5	6.8	6.8	6.6
8.3	7	6.9	6.9	6.7	7	7.2	7.3
8.3	6.7	6.7				7.1	7.3

Temperature (°C)

20.5	28	18.1	46.8	19.9	19.2	37.4	43.2
		47.8	20.1	37.1			
44		51.8	26.1	21.2	30	24.9	28.2
						23.9	20.2
29.1	20.3	46.6	27.4	29.1	29.6	18.1	46.6
				20.4	29.1	45.6	30.1
17.9	48.1	46.2	29.2	29.1	45.3	47.4	26.7
47.3	46.8	20.3				20.7	46.9

Moisture (%)

41.6	30.8	62.9	31.5	34.3	30	42.2	36.1
		27.2	60.6	23.1			
41		10.6	19.1	65.7	52	53.8	44.7
						49.4	49.7
30.1	35.3	25.5	27.2	39.5	39.5	75	48.1
				51.2	39.2	39.9	51.5
56.7	27.2	28.9	34.9	44.5	32.7	36.5	42.3
26.5	27.2	54.8				51.3	33.4

Org C (%)

0.14	0.08	0.07	0.07	0.1	0.005	0.11	0.07
		0.08	0.1	0.13			
0.08		0.14	0.08	0.09	0.09	0.08	0.07
						0.1	0.1
0.09	0.07	0.07	0.08	0.04	0.08	0.05	0.11
				0.05	0.07	0.09	0.13
0.05	0.07	0.03	0.08	0.06	0.04	0.09	0.07
0.06	0.07	0.06				0.08	0.13

Nitrate (μg g^{-1})

36.6	11.4	16.9	8.8	62.8	37.6	46.4	26.8
		35.8	7.5	66.5			
8		91.5	25.2	10.3	4.7	15.8	29.9
						6.9	14.7
5.4	7.1	13.9	11.2	11.3	18.5	4	62.1
				13.4	25.1	29	37.2
6.7	7.2	16.8	10.8	48.7	8.5	4.2	5.2
7.9	5.4	11.6				7.1	5.7

Ammonium (μg g^{-1})

8.7	8.3	5.9	8.9	6.2	7.7	8	6.7
		9.3	4.6	9.5			
5.4		8.3	7.4	7.2	7.5	7.3	7.9
						5.6	8.7
8.5	5.8	7	8.4	8.6	9.1	9.3	8
				10.7	7.3	6.6	9.4
13.8	9.7	7.8	6	8.8	8.9	5.8	7.5
6.7	6.1	5.1				8.3	7.1

Phosphate (μg g^{-1})

8.3	10.3	8.6	7.2	8.2	4.2	10.1	9.7
		7.3	8.7	5.9			
7.6		8.4	6.6	8.7	8.2	10.6	9.9
						11.9	8.7
9.3	6.9	3.8	9.7	6.8	8.6	11.8	9.6
				6.5	6.8	3.3	13.3
10.5	9.8	8.5	8.1	3.1	7.3	7.4	12.7
9.8	10.2	6.2				12.9	12.5

Magnesium (μg g^{-1})

146	85.3	65.8	88.8	129.5	146.5	130.6	107.7
		105.5	64.7	135.1			
73.5		244.5	101.6	67.1	106	93	106.4
						83.8	93.6
65.1	73.9	85.9	66.5	68.8	80.1	62	134.7
				68.6	81.8	121.7	109.1
74.5	61.6	72.4	80.9	135.2	67.9	83.2	60.9
56.9	62.8	72				63.4	65.1

Sodium (μg g^{-1})

1319 .4	784	358.2	566.2	924.2	1103.2	3659.4	2303.4
		1736.4	550.7	4117.4			
687.9		5503.4	1673.2	448.4	1415.8	1048.4	1762.4
						926	656
211.7	376.8	911.8	879.1	741.6	911.4	384	2435.4
				893	988.4	1411.4	2055.4
329.6	196.6	718.2	862.2	2699.4	482.3	801.2	324.9
37.7	234.5	866.6				334.8	309.2

Figure S1. Results from selected abiotic factors measured during diurnal study. The black boxes indicate the location of samples taken at each time-point. The red box indicates the highest reading recorded, while the orange box indicates the lowest.

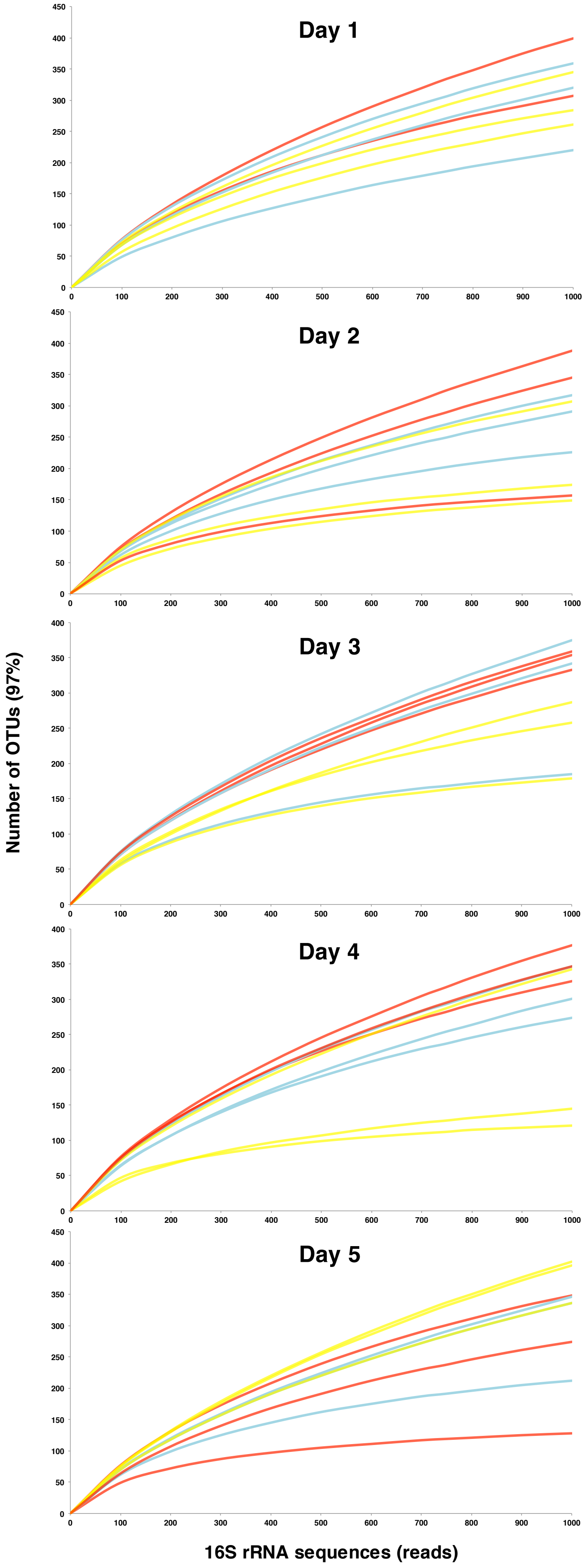

Figure S2. Rarefaction curves for bacterial communities in desert soil. Plots are shown for each day with morning (red), midday (blue) and night (yellow) highlighted.

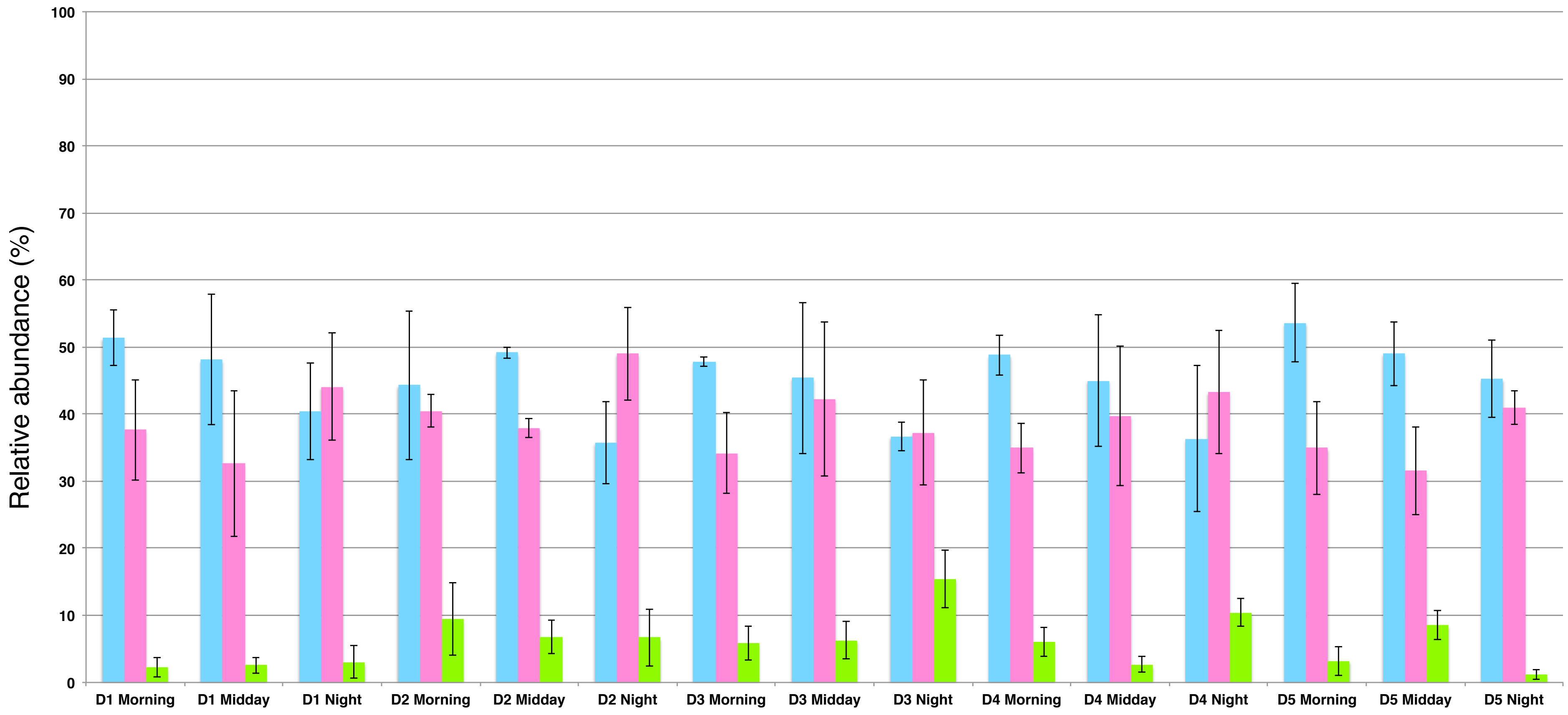

Figure S3. Color-coded bar plot showing the relative abundance of the three main bacterial phylum (Actinobacteria – blue, Proteobacteria – pink, Cyanobacteria – green)in Namib Desert soil over five diel cycles. The error bars represent the standard deviation of three biological replicates.

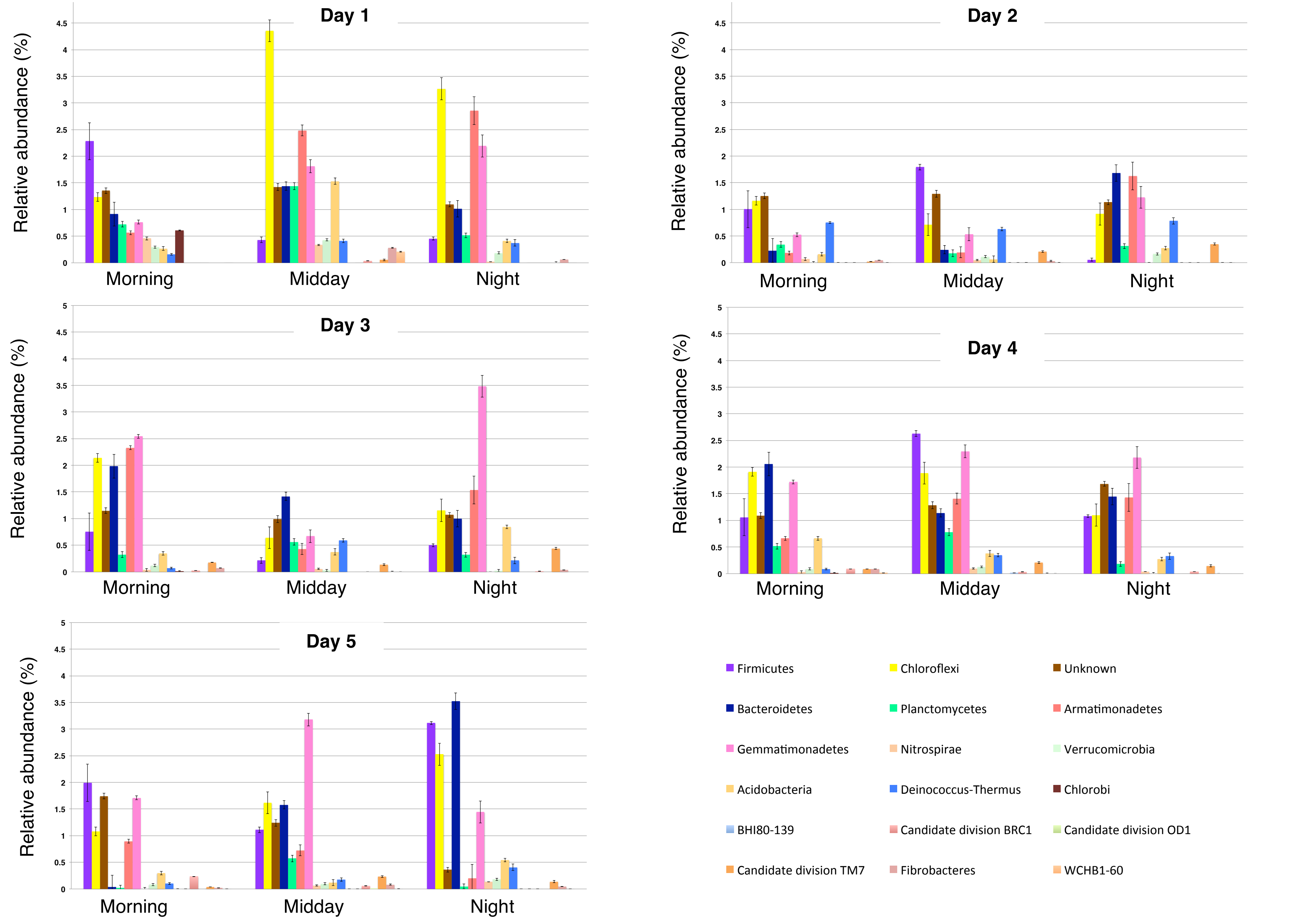

Figure S4. Color-coded bar plots showing the relative abundance of the rare bacterial phylum in Namib Desert soil over five diel cycles. The error bars represent the standard deviation of three biological replicates.

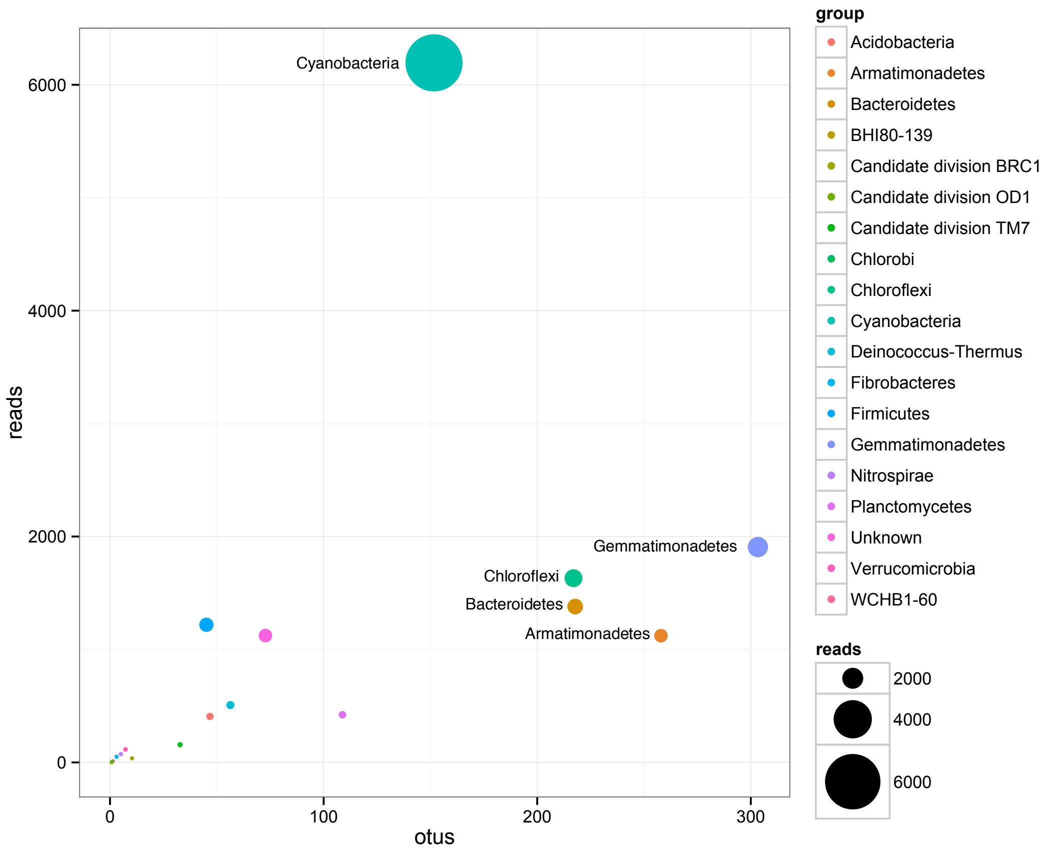

Figure S5. Bubble plot of bacterial abundances (that is, 16S rRNA gene copy numbers) without Actinobacteria and Proteobacteria.

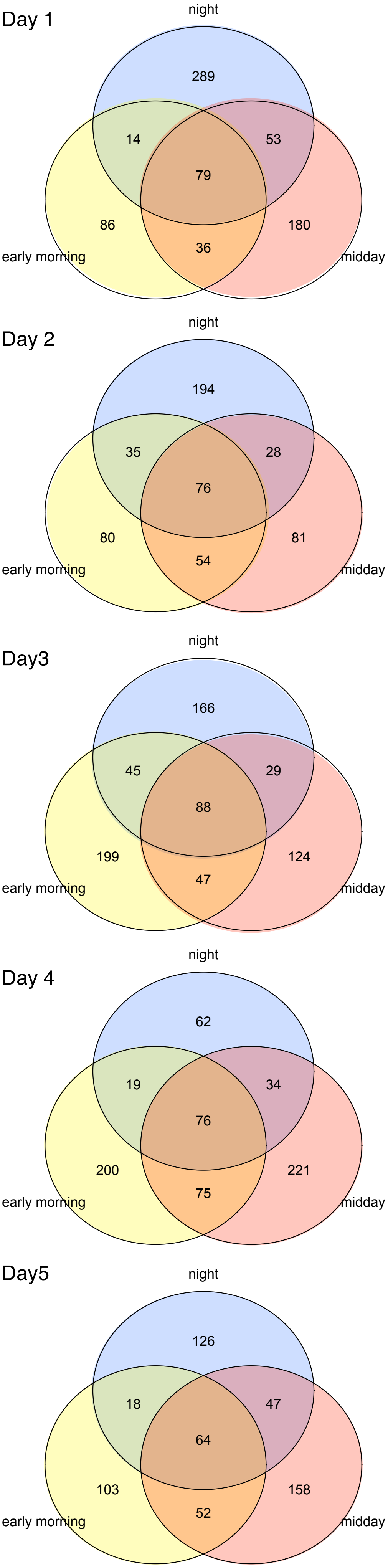

Figure S6. Venn diagrams representing the dispersal of bacterial OTUs each day. The data was resampled to lowest level (>14,000 sequences, 1688 $OTUs_{0.03}$)

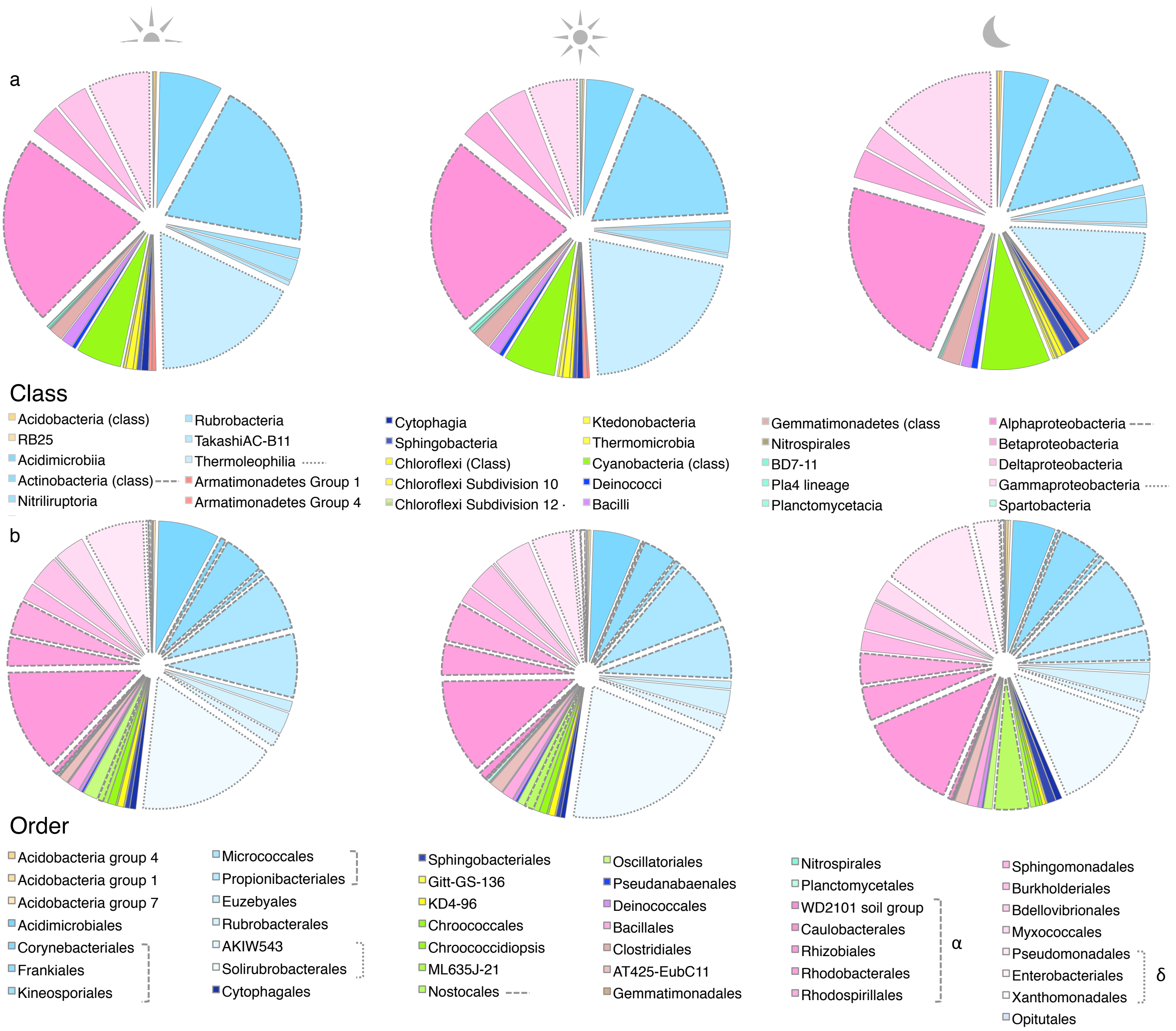

Figure S7. Phylogenetic information (relative abundance) for the pyrosequencing data at each time-point

Table S1. Mean values of soil chemistry variables measured at specific time-points over the sampling period (Morning – 6am; Midday – 1pm; Night – 8pm)

Day	Time-point	pH	Org C (%)	NH_4 (µg g-1)	NO_3^{2-} (µg g-1)	P (µg g-1)	Ca (µg g-1)	K (µg g-1)	Mg (µg g-1)	Na (µg g-1)	S (µg g-1)	CEC[c] ($cmol^+ kg^{-1}$)
**1**	**Morning**	7.63 (0.58)	0.057 (0.1)	9.7 (3.98)	9.2 (6.83)	10.29 (1.61)	2446.04 (551.56)	484.29 (167.79)	67.45 (6.44)	357.26 (27.21)	68.01 (17.32)	4.86 (0.70)
	**Midday**	6.89 (0.51)	0.076 (0.14)	7.26 (1.68)	9 (4.85)	6.13 (2.04)	3174.04 (211.06)	412.53 (77.96)	85.96 (2.76)	759.72 (176.49)	181.25 (70.17)	5.34 (0.09)
	**Night**	7.3 (0.24)	0.08 (0.12)	8.14 (0.51)	7.28 (3.41)	10.61 (1.85)	2623.04 (282.11)	317.99 (57.24)	64.18 (2.95)	471.89 (157.15)	152.18 (27.6)	4.73 (0.75)
**2**	**Morning**	7.2 (0.46)	0.109 (0.27)	7.39 (1.25)	39.08 (23.53)	9.05 (1.37)	3048.37 (212.99)	431.13 (29.81)	122.86 (27.14)	1097.59 (201.87)	159.44 (54.84)	5.18 (0.29)
	**Midday**	6.75 (0.21)	0.107 (0.31)	8.67 (0.93)	53.6 (42.75)	9.26 (0.74)	3115.37 (769.29)	403.29 (153.68)	146.97 (92.07)	2711.79 (664.17)	105.36 (31.32)	4.24 (0.06)
	**Night**	6.95 (0.35)	0.079 (0.03)	7.88 (0.47)	22.19 (9.65)	8.91 (2.04)	3157.37 (205.07)	445.69 (97.7)	97.8 (11.08)	1406.52 (540.97)	194.65 (115.44)	4.38 (0.29)
**3**	**Morning**	7.35 (0.43)	0.08 (0.05)	7.08 (1.23)	8.16 1.81)	9.51 (3.06)	2679.04 (438.33)	303.13 (19.62)	68.13 (5.3)	386.66 (57.43)	121.78 (26.86)	4.5 (0.19)
	**Midday**	7.25 (0.93)	0.06 (0.29)	7.96 (1.31)	20.16 (14.27)	8.53 (1.26)	2538.7 (645.78)	300.93 (92.84)	78.38 (24.85)	830.76 (254.93)	99.52 (35.55)	4.79 (0.8)
	**Night**	7.34 (0.79)	0.065 (0.24)	8.31 (0.71)	21.57 (13.69)	6.07 (2.63)	3292.37 (198.32)	424.63 (118.53)	103.37 (33.29)	1618.92 (664.58)	172.61 (107.37)	6.9 (2.43)
**4**	**Morning**	7.97 (0.96)	0.069 (0.55)	5.97 (1.58)	17.31 (7.54)	8.28 (3.83)	2842.04 (569.59)	447.83 (149.21)	98.36 (42.81)	859.89 (282.02)	142.18 (32.92)	4.9 (0.83)
	**Midday**	6.76 (0.74)	0.093 (0.48)	8.04 (0.91)	20.2 (12.71)	9.95 (2.49)	2693.37 (514.83)	389.26 (127.26)	87.88 (37.06)	1483.62 (686.25)	151.38 (61.54)	4.76 (0.8)
	**Night**	7.01 (0.29)	0.08 (0.05)	7.31 (1.6)	18.74 (7.99)	8.81 (0.78)	3060.37 (121.34)	429.53 (68)	89.6 (15.72)	1358.99 (818.24)	157.79 (60.42)	4.41 (0.37)
**5**	**Morning**	7.13 (0.42)	0.072 (0.31)	8.14 (2.83)	13.23 (1.55)	7.12 (1.36)	3298.04 (158.56)	415.83 (131.87)	78.06 (13.55)	805.19 (129.88)	213.25 (50.82)	4.37 (1.07)
	**Midday**	7 (0.35)	0.082 (0.12)	6.02 (0.58)	14.18 (12.9)	7.05 (3.49)	3246.04 (367.02)	433.79 (211.92)	86.03 (31.38)	777.92 (193.59)	133.98 (67.95)	4.87 (0.37)
	**Night**	6.59 (0.28)	0.111 (0.32)	8.75 (1.21)	42.95 (21.27)	8.33 (3.43)	2814.37 (420.92)	429.86 (27.58)	108.71 (26.66)	2387.06 (590.64)	139.01 (16.32)	4.56 (0.13)

Values are the mean of three biological replicates
Standard deviation in parenthesis
[c]Cation exchange capacity

Table S2. PERMANOVA table (97% cutoff) using pyrosequencing data with model time*day and contrast vales for each day and time-point measured

**Community**	**Days**			**Time-points**			**Day*time**		
**97% cutoff**	**DF**	**F**	**P**	**DF**	**F**	**P**	**DF**	**F**	**P**
Original (4348 OTUs)	4,44	1.21	0.11	2,43	1.31	0.09	8,43	1.38	<0.01
Morning vs Midday	1,44	1.23	0.17	1,43	0.67	0.93	*	*	*
Morning vs Night	1,44	0.88	0.57	1,43	1.50	0.04	*	*	*
Midday vs Night	1,44	1.11	0.25	1,43	1.40	0.10	*	*	*
Subsampled (1702 OTUs)	4,44	1.12	0.13	4,43	1.33	0.04	8,43	1.33	<0.01
Morning vs Midday	1,44	1.18	0.16	1,43	0.85	0.68	*	*	*
Morning vs Night	1,44	0.95	0.50	1,43	1.50	0.04	*	*	*
Midday vs Night	1,44	1.04	0.33	1,43	1.34	0.08	*	*	*
Subsampled, no rare (509 OTUs)	4,44	1.13	0.20	4,43	1.38	0.05	8,43	1.37	<0.01
Morning vs Midday	1,44	1.24	0.13	1,43	0.85	0.65	*	*	*
Morning vs Night	1,44	0.92	0.53	1,43	1.55	0.04	*	*	*
Midday vs Night	1,44	1.05	0.34	1,43	1.37	0.10	*	*	*

Table S3. Selection of results from correlation networks for top 100 OTUs (Sprearmens correlation coefficient [ρ] >0.6, *P*-value <0.01)

		**Intra-phyla co-occurrence (%)**			**Total positive co-occurrence (%)**			**Total negative co-occurrence (%)**		
	**Number of OTUs**	**Morning**	**Midday**	**Night**	**Morning**	**Midday**	**Night**	**Morning**	**Midday**	**Night**
Acidobacteria	1	/	/	/	12	6	5	1	1	1
Actinobacteria	47	43	57	46	147 (**46**)	190 (**64**)	164 (**46**)	21 (**38**)	107 (**50**)	33 (**36**)
Bacteroidetes	2	28	/	/	6 (**33**)	5 (0)	/	1 (0)	/	/
Chloroflexi	1	/	/	/	5	2	/	/	/	/
Cyanobacteria	5	/	13	18	16 (0)	5 (0)	9 (**44**)	2 (0)	10 (**20**)	2 (0)
Firmicutes	2	/	/	/	12 (0)	5 (0)	14 (0)	2 (0)	5 (0)	3 (0)
Gemmatimonadetes	1	/	/	/	4	4	/	/	2	/
Proteobacteria	35	49	21	45	126 (**25**)	81 (**27**)	115 (**38**)	20 (**40**)	66 (**15**)	41 (**58**)

[a] Percentage of intra-phyla correlations between OTUs
